# A Peptidic Unconjugated GRP78/BiP Ligand Modulates the Unfolded Protein Response and Induces Prostate Cancer Cell Death

**DOI:** 10.1371/journal.pone.0045690

**Published:** 2012-10-01

**Authors:** Danilo Maddalo, Antje Neeb, Katja Jehle, Katja Schmitz, Claudia Muhle-Goll, Liubov Shatkina, Tamara Vanessa Walther, Anja Bruchmann, Srinivasa M. Gopal, Wolfgang Wenzel, Anne S. Ulrich, Andrew C. B. Cato

**Affiliations:** 1 Institute of Toxicology and Genetics, Karlsruhe Institute of Technology, Eggenstein-Leopoldshafen, Germany; 2 Institute of Organic Chemistry, Karlsruhe Institute of Technology, Eggenstein-Leopoldshafen, Germany; 3 Institute of Biological Interfaces 2, Karlsruhe Institute of Technology, Eggenstein-Leopoldshafen, Germany; 4 Institute of Nanotechnology, Karlsruhe Institute of Technology, Eggenstein-Leopoldshafen, Germany; Duke University Medical Center, United States of America

## Abstract

The molecular chaperone GRP78/BiP is a key regulator of protein folding in the endoplasmic reticulum, and it plays a pivotal role in cancer cell survival and chemoresistance. Inhibition of its function has therefore been an important strategy for inhibiting tumor cell growth in cancer therapy. Previous efforts to achieve this goal have used peptides that bind to GRP78/BiP conjugated to pro-drugs or cell-death-inducing sequences. Here, we describe a peptide that induces prostate tumor cell death without the need of any conjugating sequences. This peptide is a sequence derived from the cochaperone Bag-1. We have shown that this sequence interacts with and inhibits the refolding activity of GRP78/BiP. Furthermore, we have demonstrated that it modulates the unfolded protein response in ER stress resulting in PARP and caspase-4 cleavage. Prostate cancer cells stably expressing this peptide showed reduced growth and increased apoptosis in *in vivo* xenograft tumor models. Amino acid substitutions that destroyed binding of the Bag-1 peptide to GRP78/BiP or downregulation of the expression of GRP78 compromised the inhibitory effect of this peptide. This sequence therefore represents a candidate lead peptide for anti-tumor therapy.

## Introduction

The glucose regulated protein GRP78 (also known as BiP, immunoglobuling heavy chain binding protein) is a member of the heat shock protein family and plays an important role in maintaining cellular homeostasis [1]. It is the key regulator of the unfolded protein response (UPR), a pathway activated upon accumulation of unfolded peptides during stressful conditions such as heat shock, acidosis, nutrient starvation and hypoxia [2].

GRP78 regulates the UPR by binding the transmembrane sensor proteins PERK (PKR-like endoplasmic reticulum-resident kinase), ATF6 (activating transcription factor 6) and IRE1α (inositol-requiring enzyme α) (reviewed in [3]) leading on the one hand to an increased transcription of molecular chaperones like GRP78 itself, GRP94 and protein-disulfide isomerase (PDI) [4], [5] and on the other hand to protein synthesis shutdown by phosphorylation of the alpha subunit of the eukaryotic initiation factor eIF2α [6]. As a consequence of these two effects, cells overcome being overloaded with aberrant peptides and they survive [7]. However, prolonged eIF2α phosphorylation activates the transcription factor ATF4 [8], [9] leading to increased levels of the pro-apoptotic factor CHOP (C/EBP homologous protein) [10], [11]. Activation of ER-stress mediated apoptosis results in cleavage of caspsase 4, an ER-stress specific caspase, and of PARP (poly(ADP)-ribosome polymerase) [12], [13].

GRP78 is overexpressed in several types of tumors such as prostate [14], breast [15], [16] and colon and its expression often correlates with poor prognosis [17], [18], [19]. However GRP78 downregulation by siRNA increases apoptosis and sensitizes cells to chemotherapeutic drugs [20], [21]. In general transformed cells upregulate GRP78 level [15] to survive the adverse conditions of the tumor microenvironment [22], [23], [24]. Several therapeutic agents have therefore been targeted against the UPR or against GRP78/BiP to curb tumor cell growth [25], [26] but truly selective inhibitors are yet to be identified [15]. In a search for further inhibitors of GRP78/BiP that would be of therapeutic relevance, we have used information on the regulation of ER stress by the cochaperone Bag-1 [27] to identify a sequence from Bag-1 that binds to and inhibits the action of GRP78/BiP.

Bag-1 is a family of four polypeptides (Bag-1L, -1M, -1 and -1S) with multifunctional domains that interacts with and regulates the activities of diverse cellular proteins [28]. These proteins possess divergent N-terminal sequences but a common centrally located ubiquitin-like domain that forms a link for Hsc/Hsp70 to the proteasome [29] and a conserved C-terminal Hsp70 binding domain (otherwise known as the BAG domain) that binds to Hsp70/Hsc70 and functions as a nucleotide exchange factor [30], [31]. Bag-1 has also been shown to regulate endoplasmic reticulum (ER) stress-induced apoptosis [27] and to bind GADD34, a component of the ER stress [32] but details of its action are not known.

In this communication we show that Bag-1 binds to GRP78/BiP through a peptide overlapping its ubiquitin-like domain. We further show that the GRP78/BiP binding peptide of Bag-1 inhibits the action of GRP78/BiP and interferes with the UPR leading to the induction of apoptosis. We have narrowed down this peptide and identified a core motif of seven amino acids that appears essential for binding to GRP78/BiP and for the negative regulation of prostate tumor cell growth. This core sequence could be the starting point of future therapeutics directed towards the inhibition of GRP78/BiP action and of the UPR.

## Materials and Methods

### Cell Culture

Human benign prostatic hyperplasia cell line BPH-1 was cultured in Dulbeccós modified Eaglés medium (DMEM) supplemented with glutamine. PC3 and DU145 cells were also cultured in DMEM but without glutamine 22Rv.1, LNCaP and PNT-2 cells were cultured in RPMI 1640. All the above culture media were supplemented with 10% fetal bovine serum. RWPE-1 cells were cultured in keratinocyte serum free medium. All the culture media were kept at 37°C in an atmosphere of 5% CO_2_.

### Antibodies

Goat monoclonal antibody GRP78 (N20), rabbit polyclonal antibodies against Bag-1 (C-16), eIF2α and phospho-PERK were purchased from Santa Cruz Biotechnology, Heidelberg, Germany. Rabbit polyclonal anti-GRP78 (ab21685), anti-GCN2 (ab37674), anti-PERK (ab65142), anti-phospho-IRE1α (ab124945) antibodies, rabbit monoclonal anti-phopsho-GCN2 (ab75836) antibody and mouse monoclonal anti-β actin (ab8226) antibody were purchased from Abcam, Cambridge, UK. Mouse antibody against HA-tag (HA.11 clone 16B12) was purchased from Covance, Munich, Germany. Rat monoclonal antibody against HA (3F10) was purchased from Roche, Mannheim, Germany. Antibodies against IRE1α, phospho-eIF2α CHOP, PARP and ATF4 were purchased from Cell Signaling Technology, Frankfurt am Main, Germany. Anti-ATF6 antibody was purchased from Imgenex, Hamburg, Germany. Caspase 4 antibody was purchased from MBL, Munich, Germany.

### Expression Vectors and Plasmids

The expression vector pcDNA3.1-HA-Bag-1 encoding Bag-1 has been previously described by [33]. The construct encoding Bag-1Δ68mer (Bag-1 lacking amino acids 202–269) was created by replacement of Sac II-Xba I fragment of the Bag-1 cDNA in a pcDNA3-Bag-1 construct. The construct pcDNA3-HA Bag-1 peptide (Bag1-L 202–269), N-terminal (Bag-1L 202–241), C-terminal (Bag-1L 241–269), 19-mer (Bag-1L 202–220), ΔUbi (Bag-1L 220–269) and 19-mer mutant (Bag-1L 202–220) peptides were created by PCR amplification with an HA sequence. As a control, we introduced the HA sequence into pcDNA3 vector to generate the empty expression vector pcDNA3.1-HA. pcDNA3.1 Bag-1ΔC47 (Bag-1 lacking the last 47 amino acids) was cloned into the Bam HI-Xho I sites of pcDNA3.1-HA vector after PCR amplification using pcDNA3.1-HA Bag-1 as template. For GST-pull-down experiments, pGEX4T.1-Bag-1 68mer sequence, pGEX4T.1-N-terminal peptide, pGEX4T.1-C-terminal peptide, pGEX4T.1 Bag-1Δ Ubi, pGEX4T.1-19mer and pGEX4T.1-19mer mutants were created by fusing PCR amplification products of the respective Bag-1L sequence in frame with GST in the vector pGEX4T.1. GST plasmids encoding for GST-fused GRP78, GRP78-ATPase (GRP78 1–408), GRP78-SBD (GRP78 409–651) and Bag-1 isoforms were generated by PCR and cloned into pGEX4T.1 after BamHI-XhoI digestion. The plasmid pCMV6-GRP78 [34] was purchased from OriGene Technologies (Darmstadt, Germany).

### Cell Transfection, siRNA Knock-down and Western Blotting

Unless otherwise stated, stable transfection was carried out in 22Rv.1 or BPH-1 cells with 10 µg of plasmid DNA using FuGene 6™ (Roche Diagnostics Mannheim, Germany) Stably transfected 22Rv.1 cells were selected with 0.8 mg/ml while BPH-1 cell clones were selected with 1.2 mg/ml G418. LNCaP, PC3, DU145, PNT-2 and RWPE-1 cell clones were selected with 1 mg/ml G418. Specific downregulation of GRP78 expression was achieved by transfecting siRNA-duplexes (GRP78∶5′-GGAGCGCAUUGAUACUAGATT-3′) twice in 72 h using HiPerfect™ (Qiagen Hilden, Germany). siRNA against GFP (5′-GGCUACGUCCAGGAGCGCACC-3′) was used as a control. Western blot analysis was performed as previously described [35].

### Co-immunoprecipitation

22Rv.1 cells were used for interaction studies of endogenous Bag-1 and GRP78/BiP, while for the *in vivo* interaction of the Bag-1 peptide and GRP78/BiP, HEK-293 cells were transfected with a plasmid encoding HA-tagged Bag-1 peptide. Twenty-four hours prior to the experiment, the cells in both cases were washed with phosphate buffered saline (PBS) and treated with 20 mM Dithiobis(succinimidyl propionate) (DSP) in PBS for 30 min at room temperature. The reaction was stopped with 20 mM Tris and the cells were lysed in 50 mM Tris-HCl pH 7.4, 120 mM NaCl, 1 mM EDTA, 0.4% NP40 and 1% protease inhibitor cocktail. Immunoprecipitation of GRP78/BiP was carried out overnight at 4°C with anti-GRP78 antibody coupled to sepharose A beads (Abcam, ab21685). Binding of endogenous Bag-1 or of HA-peptide was determined by Western blotting using a Bag-1 or an HA specific antibody.

### Protein Expression and Sample Preparation

A TEV protease-cleavable GB1-fused Bag-1 peptide was expressed in the *Escherichia coli* strain BL21 (DE3) pLys S. Uniform labeling with ^15^N for NMR spectroscopy was achieved by growing the bacteria in minimal medium supplemented with 0.5 g/l ^15^NH_4_Cl. The GB1-His_6_-tagged protein was purified over a Ni-NTA column (Qiagen, Hilden, Germany), subsequently digested with recombinant TEV protease and passed over a second Ni-NTA column to remove both the fusion tag and the His_6_-tagged protease. Finally, the protein buffer was changed to 20 mM potassium phosphate, 100 mM NaCl, pH 6.8. Protein concentration was estimated by comparing the intensity of a 1D NMR spectrum with that of a reference substance (DSS, 2,2-dimethyl-2-silapentane-5-sulfonic acid).

The GST-HA-tagged N-terminal and C-terminal peptides were produced using standard protocol. The GST moiety was cleaved off by digestion with thrombin according to the manufactureŕs protocol (Roche, Mannheim, Germany). The resulting crude peptide preparation was further purified by reversed phase HPLC (C18 column; 250×10 mm, Grace) with a water/acetonitrile linear gradient. The identity and purity of the collected fractions were analysed by MALDI-TOF mass spectrometry (Bruker Autoflex III). The pH was neutralized prior to lyophilization. The peptide was dissolved in 20 mM KPO_4_, pH 6.8. Protein concentration was calculated from the optical density at 275 nm using the absorbance of the tyrosines in the HA-tag.

### CD Spectroscopy

The CD spectra of the peptides were recorded on a Jasco J-810 spectropolarimeter (Jasco Co., Tokyo, Japan) at 20°C, using a water-thermostated cell holder. The concentrations of the Bag-1, N-terminal and C-terminal peptides were 17 µM, 12 µM, and 11 µM, respectively. The CD spectra are averages of three scans at a scan rate of 10 nm min^−1^, 4 s response time and 1 nm band width, and they were smoothened by the adaptive smoothing method that is part of the Jasco Spectra Analysis software. The buffer contribution was subtracted.

### NMR Experiments

Protein concentration for the NMR experiment was 0.5 mM. For chemical shift calibration and to compare relative signal intensities, 0.2 mM DSS (2,2 Dimethyl-2-silpentane-5-sulfonic acid) was added. ^15^N-HSQC spectra were acquired at 23°C on a Bruker Avance II 600 spectrometer (Bruker, Karlsruhe) equipped with a broadband triple resonance probe head with 4 scans per increment and a total of 128 increments in the indirect dimension. Data were processed with NMRPIPE [36] and analyzed using NMR VIEW.

### Fluorescence Polarization Assays

Fluorescence polarization assays were carried out using an Infinite F-200 reader (Tecan) with excitation at 490 nm and emission at 535 nm.

### Tumor Xenograft Studies

All animal experiments were performed according to European and German statutory regulations. For generation of xenograft models 5×10^6^ LNCaP cells were resuspended in a solution of 50% Matrigel® (BD Bioscience, Heidelberg, Germany) in PBS or 5×10^6^ 22Rv.1 cells in 100 µl of PBS were subcutaneously injected into both flanks of 6–8 week-old athymic nude mice. Tumor size was measured with a caliper every week. Tumors derived from the injection of stable clones overexpressing the Bag-1 peptide were analyzed over a period of 9 weeks while tumors from cells transfected with the empty expression vector or expressing the N-terminal, the C-terminal and the ΔN-peptide were analyzed over 5 weeks. Tumor size was assessed measuring three perpendicular diameters according to the formula: V = (1/6) [π] (d1d2d3), where π is a mathematic constant and d1, d2 and d3 represent the three spatial dimensions (width, depth and height). Mice were euthanized by cervical dislocation and the tumors removed for further analysis.

### GST-pull-down Experiments

Expression of GST fusion proteins for GST-pull-down experiments were performed essentially as described previously [37].

### Immunohistochemistry

Tissues were fixed in 10% formalin for 16 h at room temperature, stored in ethanol (50%), paraffin embedded, and sectioned 5 µm thick with a Leica RM 2155 microtome. Apoptotic cells were detected via terminal deoxynucleotidyl transferase-mediated deoxyuridine-triphosphate-biotin nick end labeling (TUNEL) assay using the DNA fragmentation ApopTag® peroxidase *in situ* apoptosis detection kit (Millipore, Schwalbach, Germany).

### Cell Viability Assay

CellTiter-Blue® cell viability assay kit (Promega) was used for the determination of cell viability. Briefly 22Rv.1 cells stably overexpressing the Bag-1 peptide were seeded into two 96 well plate (4500 cells/well) and the measurements were performed in triplicate. One plate was analyzed at day 0 and the other plate, 2 days later. Three hours after seeding, 20 µl of dye were added to each well containing 100 µl of culture medium and incubated for 4 h at 37°C. Fluorescence was measured by the plate-reading fluorometer FluoStar Optima (2001, BMG Labtechnology, software version 1.10–0) and presented as the ratio of the value at the excitation wavelength (560 nm) over the emission wavelenght (590 nm). Cell proliferation was determined from the difference between the fluorescence intensity at the final time point and the value obtained at the day of seeding of the cells (day 0).

### *In vivo* Refolding Assay

In vivo refolding assay was performed essentially according to the method described by [38]. HEK-293 in a 70%-confluent 10 cm dish were transfected with β-actin-fire fly luciferase construct (2 µg), pcMV6-GRP78 (3 µg), Bag-1 or Bag-1 peptides in pcDNA3.1-HA (6 µg) and Renilla luciferase construct (0.5 µg) for equal transfection control. Two days after transfection the cells were divided into two and incubated in heat shock buffer (MOPS 20 mM and cycloheximide 20 µg/ml) for 30 min at 37°C. One half of the transfected cells was heat shocked for 30 min at 45°C and allowed to recover at 37°C for 30 min. The other half was left untreated and used as control. At the end of the experiments, the cells were harvested, lysed and luciferase activity was measured. The activity measured for the non-heat shocked cells was set as 100% of refolded luciferase and the activity measurements of the heat shocked cells were expressed relative this value.

### Statistical Analysis

Unless otherwise stated calculations of statistical significance in this work was performed according to Student’s t test.

## Results

### Bag-1 Interacts with GRP78/BiP

**Figure 1:**
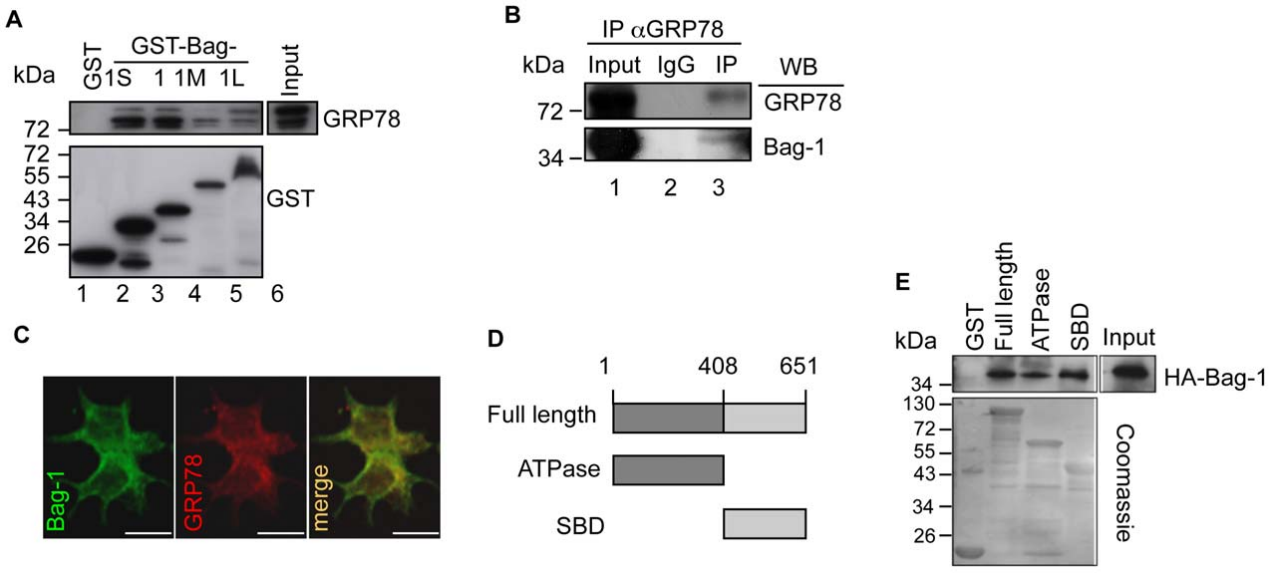
Bag-1 proteins interact with the molecular chaperone GRP78/BiP. A. The Bag-1 family of proteins binds GRP78/BiP. GST pull-down assay was performed incubating 5 µg each of GST-fused Bag-1 proteins with 400 µg of 22Rv.1 cell lysate. Western blotting with a specific antibodiy against GRP78/BiP was used to detect the binding and an anti-GST antibody was used to determine the amount of bacterially purified protein employed in the assay. B. Bag-1 and GRP78 interact *in vivo*. GRP78 was immunoprecipitated with an anti-GRP78/BiP antibody or IgG as control in 22Rv.1 cells. Western blotting with an anti-Bag-1 and anti-GRP78 antibody was performed to determine GRP78-Bag-1 interaction. C. Bag-1 and GRP78/BiP show a perinuclear colocalization. Confocal microscopic analyses were carried out in 22Rv.1 cells that have been paraformaldehyde-fixed and stained with a Bag-1 antibody to detect Bag-1 (green channel) and a GRP78/BiP specific antibody to detect GRP78/BiP (red channel). The orange staining in the merge of the two channels indicates the degree of co-localization of the two proteins. All images (40X) were acquired with a Leica TCS SPE confocal microscope (Leica Microsystems; Scale bars indicate 25 µm). D. Diagrammatic representation of the domains of GRP78. E. Bag-1 binds to multiple sites on GRP78. GST-pull down assay performed incubating 500 µg lysate of HEK-293 cells transfected with an HA-tagged Bag-1 and 10 µg GST-fused GRP78 full length and its ATPase and substrate binding domain (SBD). Western blot analysis was carried out using an HA antibody to detect the Bag-1-tagged protein. Shown is a Coomassie blue staining of the bacterially purified GST proteins to demonstrate equal loading of the GST proteins.

As Bag-1 is reported to mediate ER-stress and to interact with one of the components of the UPS [27], [32], we investigated whether it also binds to GRP78/BiP, a key component in the ER stress pathway. We first determined whether Bag-1 interacts with GRP78/BiP in a GST pull-down assay using lysate from 22Rv.1 prostate cancer cells. Western blot analysis using a GRP78/BiP specific antibody showed that all the members of the Bag-1 family of proteins interacted with GRP78/BiP (Figure 1A). *In vivo* interaction was also demonstrated in a co-immunoprecipitation assay in which Bag-1 was shown to bind to GRP78/BiP in 22Rv.1 cells (Figure 1B). Furthermore, we could show a perinuclear localization of Bag-1 and GRP78/BiP in an immunofluorescence assay using 22Rv.1 cells (Figure 1C) implying an ER localization of Bag-1. This finding was confirmed in another immunofluorescence experiment where we could show colocalization of Bag-1 with an ER tracker (Figure S1).

To determine the domain of GRP78/BiP involved in its binding to Bag-1, we used GST-fusion constructs of the two main regions of GRP78/BiP (the ATPase and substrate binding domain-SBD) (Figure 1D) in pull-down experiments with HEK-293 cells overexpressing Bag-1. Western blot analysis showed that Bag-1 interacted not only with the full length GRP78/BiP but also with its ATPase and SBD (Figure 1E). As Bag-1 is reported to bind to the ATPase binding domain of the molecular chaperone Hsp70/Hsp70 [39] and GRP78/BiP belongs to this family of chaperones [40], our finding that the SBD of GRP78/BiP is also bound by Bag-1 is rather intriguing and identifies a novel interaction site of Bag-1 in the molecular chaperone family. We therefore used the SBD of GRP78/BiP in further characterization of the interaction of GRP78/BiP with Bag-1.

**Figure 2:**
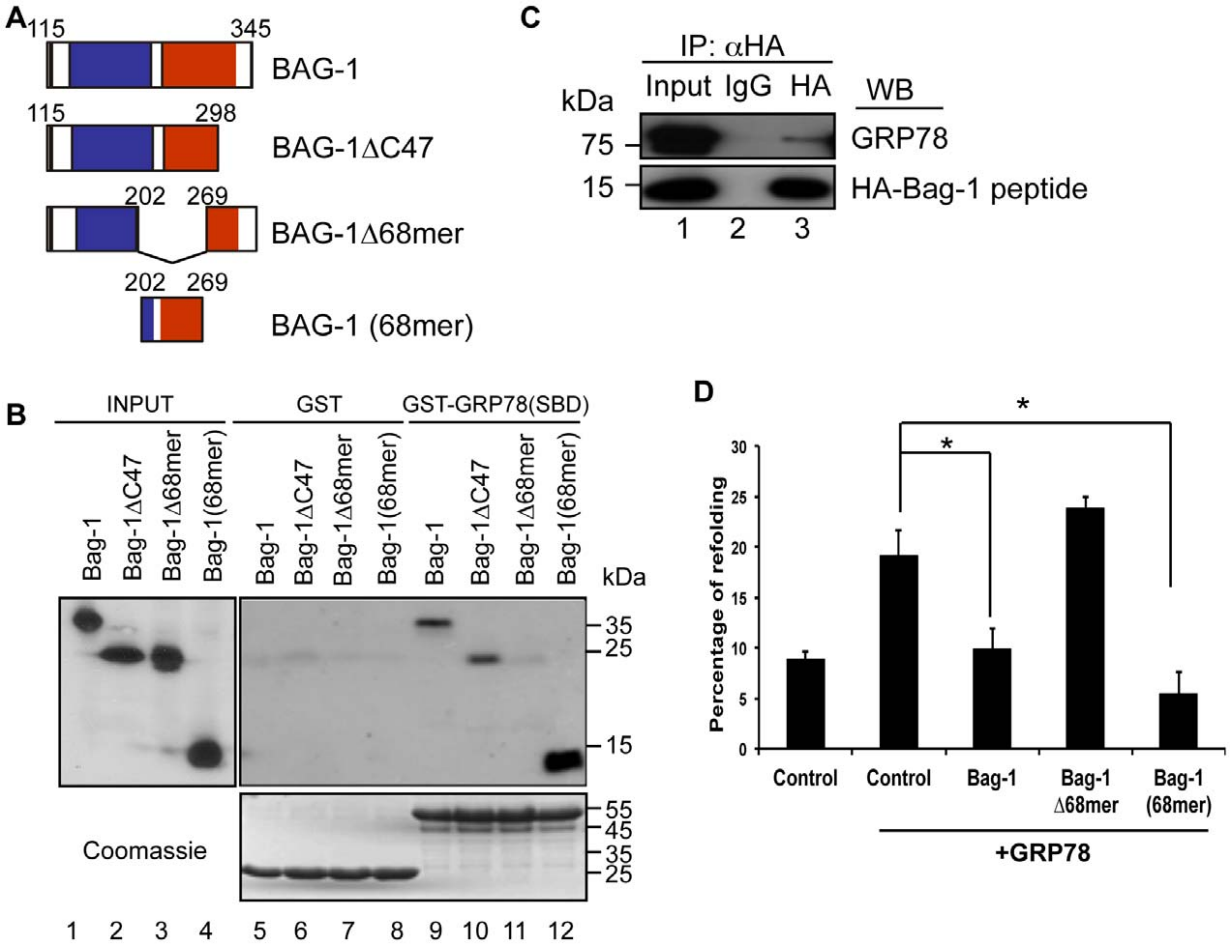
Identification of Bag-1 peptide interfering with the action of GRP78/BiP. A. Diagrammatic representation of Bag-1 and its deletion mutants used for the identification of sequences required for binding the SBD of GRP78/BiP. Depicted in blue and red are the ubiquitin-like domain and the BAG domain respectively. B. Identification of the Bag-1 peptide binding to GRP78/BiP. GST-pull down assay performed incubating 500 µg lysates from HEK-293 cells transfected with the indicated Bag-1 constructs and 10 µg of GST-GRP78(SBD) or GST as control. Western blot analysis with an HA antibody to detected the Bag-1 mutants is shown. Coomassie blue staining was performed to demonstrate equal loading of the GST fusion proteins. C. The Bag-1 68 amino acid peptide binds *in vivo* to GRP78. HEK 293 cells were co-transfected with pcDNA-HA-tagged Bag-1 peptide. The transfected cells were treated with 2 mM Dithiobis(succinimidylpropionate) to cross-link the proteins and the cell lysates were immunoprecipitated with an anti-HA antibody or IgG as control, followed by Western blotting with an anti-GRP78/BiP antibody. D. The Bag-1 68 amino acid peptide inhibits GRP78/BiP refolding activity. *In vivo* luciferase refolding assay was performed in HEK-293 cells transfected at 70% confluency with 2 µg β-actin-fire fly luciferase construct, 3 µg pcDNA3 empty vector as control or pCMV6-GRP78/Bip, 6 µg of the indicated pcDNA3.1-Bag-1 or mutant constructs and 0.5 µg Renilla luciferase construct to determine the transfection efficiency. The transfected cells were divided into two. One half was left untreated and the other half was heat shocked at 45°C for 30 min. Thereafter luciferase activity was measured. Results are expressed as percentage of refolding activity relative to non-heat-shocked cells and presented as bar charts of the average of three independent experiments ± SEM. *p<0.05.

GST-pull down analyses were carried out with the SBD of GRP78/BiP and lysate of HEK293 cell expressing the wild type Bag-1, Bag-1ΔC47, a C-terminal deletion mutant or Bag-1Δ68mer, an internal deletion mutant. These studies identified an internal sequence of 68 amino acids as a target of interaction of Bag-1 with GRP78/BiP (Figures 2A and B). Deletion of this sequence (Bag-1Δ68mer) abolished the interaction of Bag-1 with GRP78/BiP (Figure 2B lane 11) while expression of the 68 amino acid sequence alone showed that it is indeed required for binding the SBD of GRP78/BiP (Figure 2B lane 12). This finding was further confirmed in an *in vivo* co-immunoprecipitation experiment where an HA-tagged Bag-1 peptide expressed in HEK 293 cells interacted with endogenous GRP78/BiP protein (Figure 2C lane 3).

**Figure 3:**
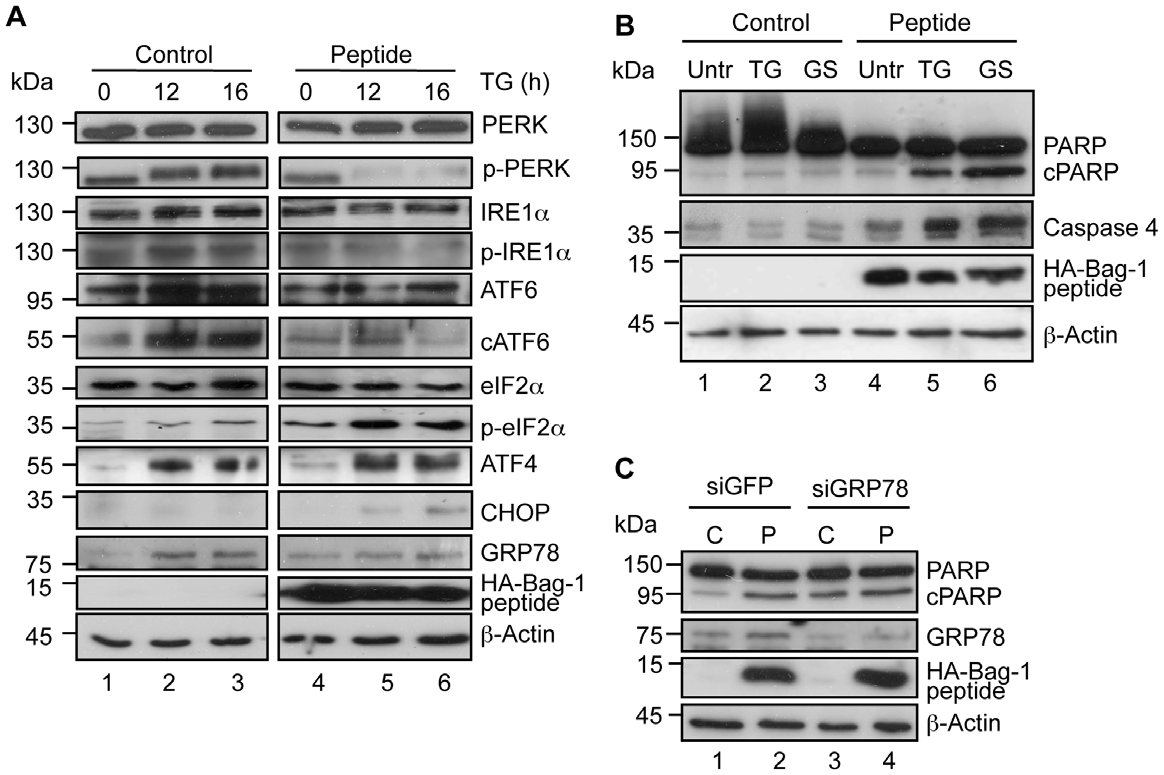
The Bag-1 peptide modulates the UPR and sensitizes 22Rv.1 cells to ER-stress. A. UPR modulation upon Bag-1 peptide overexpression. Pooled clones of 22Rv.1 cells transfected with pcDNA3.1-HA-Bag-1 68 amino acid peptide or an empty expression vector were treated with 300 nM thapsigargin (TG) for the indicated time points. Cells were lysed and subjected to Western blot analysis using the indicated antibodies or phospho-specific antibodies. B. The Bag-1 peptide sensitizes 22Rv.1 cells to ER-stress induced apoptosis. Pooled clones of 22Rv.1 transfected with the Bag-1 peptide or the empty expression vector were treated with thapsigargin (TG) or glucose-starved (GS) for 24 h. The cells were lysed and subjected to Western blot analysis using anti-PARP and caspase 4 specific antibodies. Anti-HA antibody was used to detect the HA-Bag-1 peptide. Anti-β-actin antibody was used to demonstrate equal loading of the protein samples. C. GRP78 downregulation increases PARP cleavage. Pooled clones of 22Rv.1 expressing HA-tagged Bag-1 peptide or an empty expression vector were transfected with GRP78/BiP siRNA or control GFP siRNA. The cells were lysed and Western blot was carried out with anti-PARP, anti-GRP78 and anti-HA antibodies. β-actin antibody was used to determine the level of protein loaded on the gel.

Several studies have shown that GRP78/BiP cooperates with PDI in refolding denatured proteins *in vitro* [41], [42]. To determine whether GRP78/BiP also possesses the ability to fold denatured proteins *in vivo*, we adopted a refolding assay used previously to determine the chaperone activity of Hsp70 *in vivo* [43] In this assay, HEK-293 cells were transfected with a plasmid encoding a luciferase gene and an expression plasmid for GRP78/BiP. The cells were briefly heat shocked and thereafter the luciferase activity of the transfected cells expressing GRP78/BiP was compared with that of the non-GRP78/BiP expressing cells. This study showed that overexpression of GRP78/BiP significantly enhanced luciferase activity after the heat shock (Figure 2D) demonstrating the ability of GRP78/BiP to refold denatured luciferase *in vivo*. If the cells were additionally transfected with Bag-1 or the 68 amino acid Bag-1 peptide that binds GRP78/BiP, the refolding activity of GRP78/BiP was reduced to the control level (Figure 2D). This was not the case when Bag-1Δ68mer that does not bind GRP78/BiP was cotransfected. These studies demonstrate that the Bag-1 peptide interferes with the refolding activity of GRP78/BiP. However the Bag-1 peptide did not have any effect on the ATPase activity of GRP78/BiP although Bag-1 increased this enzymatic activity (Figures S2A and S2B). This indicates that the Bag-1 peptide functions differently from the full length Bag-1. We therefore embarked on the characterization of this peptide as a ligand for GRP78/BiP for future therapeutic use.

**Figure 4:**
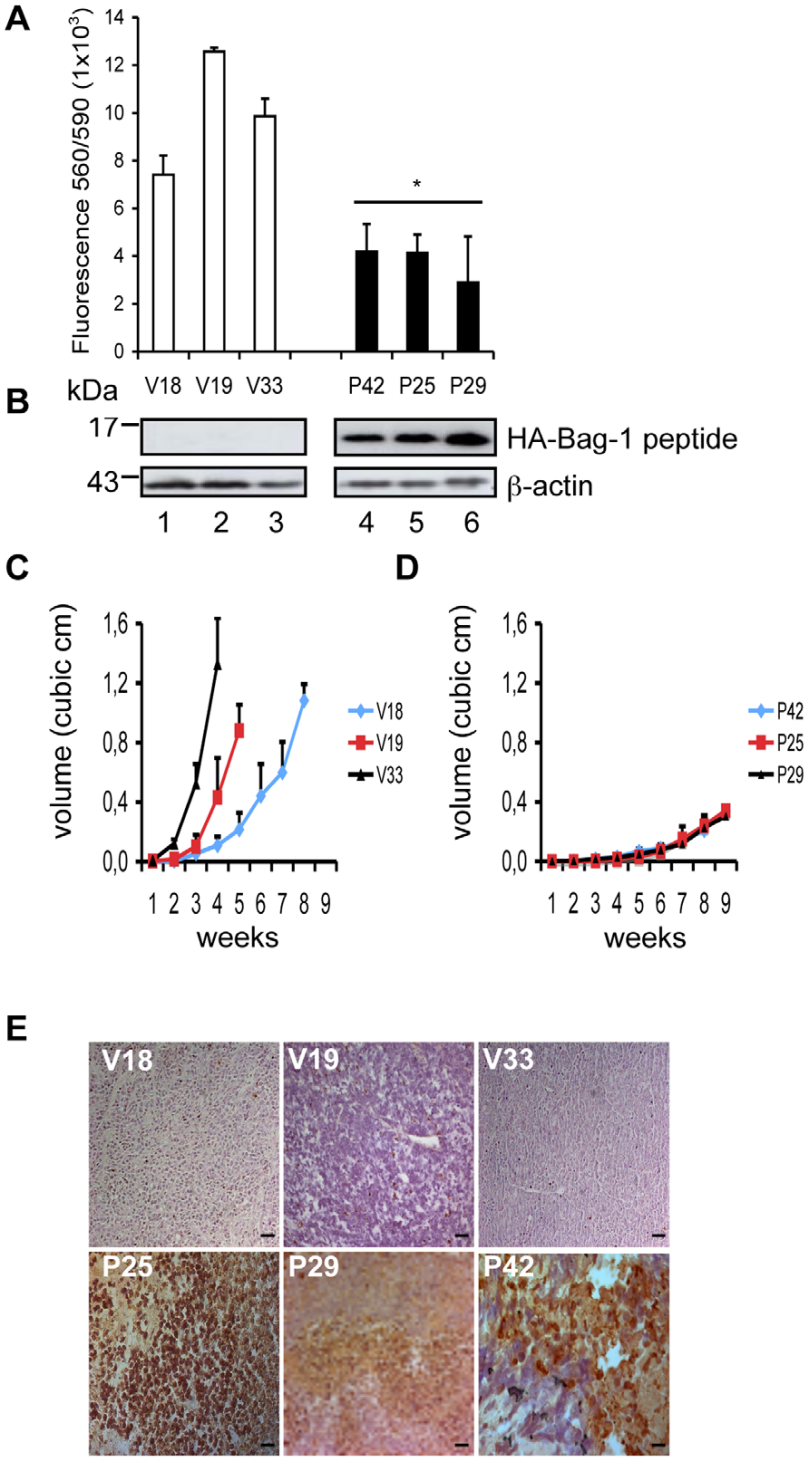
The Bag-1 peptide inhibits prostate cancer cell growth *in vivo*. A. Stable clones of 22Rv.1 cells show reduced cell viability *in vitro*. Stable clones were seeded in a 96-well plate and the cell number in each well was estimated by measuring the ratio between the wavelength of excitation and emission 560/590 nm using the CellTiter-Blue™ proliferation assay. The bar charts represent the mean of at least four independent experiments ±SD (*p<0.05). B. Determination of the level of the Bag-1 expressed in the 22Rv.1 clones. Cellular extracts of 22v.1 stable clones were subjected to Western blot analysis. An anti-HA antibody was used to detect the peptide and an anti-β-actin antibody for equal loading control. C-D. The Bag-1 peptide reduces 22Rv.1 prostate tumor cell growth *in vivo*. Six-week old athymic nude mice were injected subcutaneously in both flanks with 5×10^6^ cells of each stable clone. Tumor size was measured once per week using a caliper and expressed as tumor volume in mm^3^. Shown are (C) the tumor volumes of clones transfected with empty expression vector and (D) clones expressing the Bag-1 peptide. Each point represents the mean volume and standard deviation of at least 5 to 10 tumors. E. Xenografts of stable clones expressing the Bag-1 peptide show increased apoptosis. Five-micron sections of formalin-fixed, paraffin-embedded tumor tissues were subjected to immunohistochemistry. Nuclei are stained blue-purple with hematoxylin while apoptotic cells detected with the TUNEL assay are stained brown. Representative sections of xenografts obtained from each 22Rv.1 stable clone were acquired with an Axioscop microscope (Zeiss). V: stands for empty vector expressing clone and P: peptide expressing clone.

**Table 1:** Effect of the Bag-1 peptide on tumor weights in 22Rv.1 and LNCaP xenograft tumor mouse models.

22Rv.1 cells			
	Clone No.	Tumor weight(g)	Termination time (weeks)
**Vector clones**	V33	1.24±0.61	4
	V19	1.36±0.54	4–5
	V18	1.73±0.48	7–8
**Peptide clones**	P25	0.31±0.14	7–9
	P42	0.38±0.20	9
	P29	0.42±0.17	9
**LNCaP cells**			
	**Clone No.**	**Tumor weight** **(g)**	**Termination time (weeks)**
**Vector clones**	V82	0.65±0.29	8
	V69	0.22±0.03	6
**Peptide clones**	P35	0.05±0.01	6
	P12	0.04±0.03	8

Tumor weights and time of euthanasia of mice are recorded. The values represent the mean weights and standard deviation calculated from 4–10 tumors and the time of euthanasia.

### Utilization of a Bag-1 Peptide to Induce Apoptosis and Reduce Prostate Cancer Cell Growth

During ER stress, unfolded peptides accumulate in the ER and GRP78/BiP plays a pivotal role to adjust protein folding capacity by activating three signaling pathways (PERK, IRE1α, and ATF6) [44], [45]. PERK is autophosphorylated leading to the phosphorylation of the alpha subunit of eIF2 and protein synthesis shut-down. Phosphorylated eIF2α selectively enhances translation of the transcription factor ATF4 that increases UPR target gene expression such as GRP78/BiP [44], [45] and IRE1α is also autophosphorylated leading to the activation of chaperone synthesis via Xbp1 activation. ATF6 is proteolytically cleaved to take part in the upregulation of expression of UPR target genes [44], [45]. However apoptosis is induced if homoeostasis cannot be established [46]. We therefore determined whether binding of the Bag-1 peptide to GRP78/BiP, the key component of ER stress, affects the signaling pathways of the UPR.

**Figure 5:**
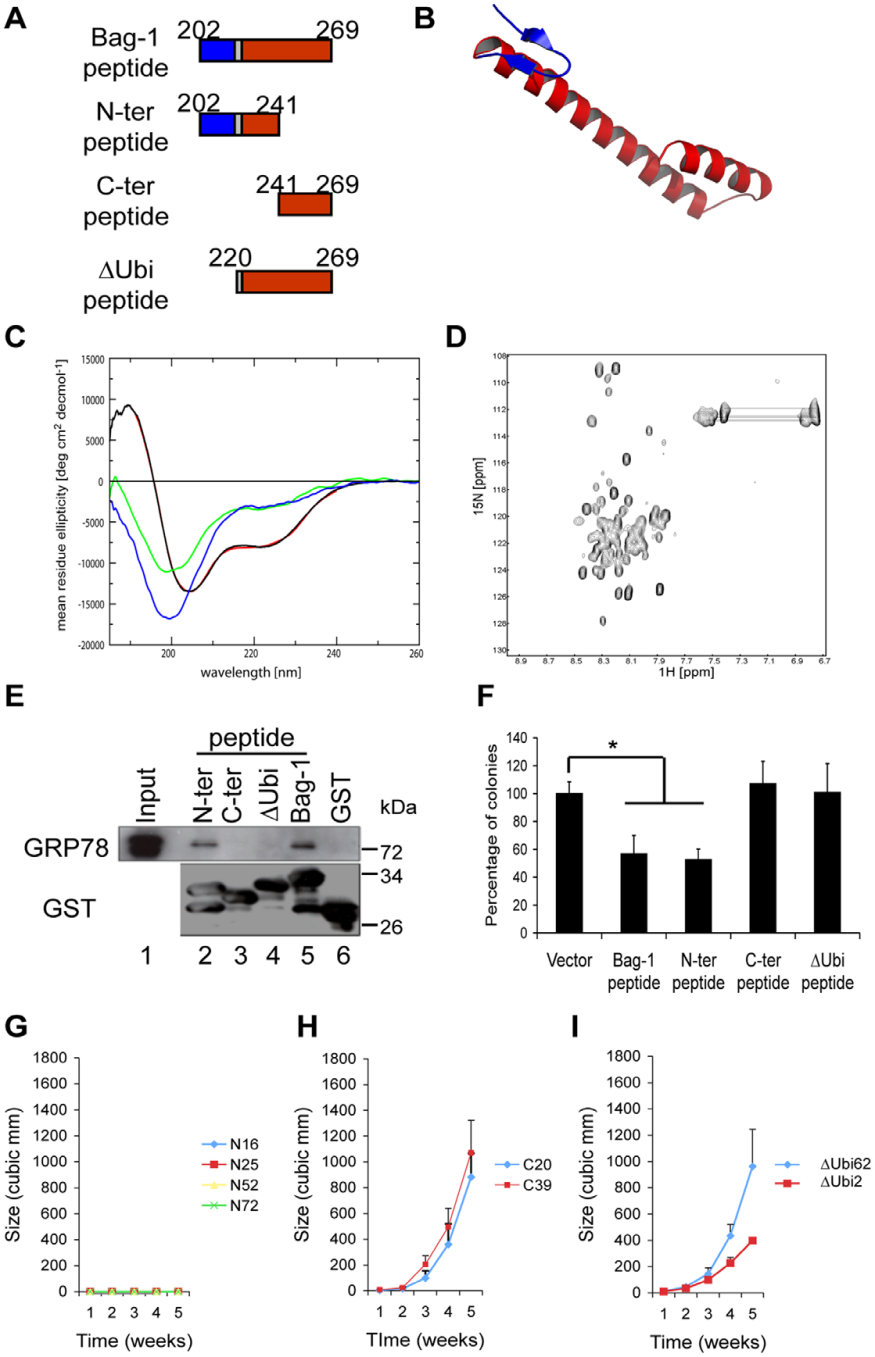
An N-terminal unfolded fragment of the Bag-1 peptide is sufficient to reduce tumor cell growth. A. Schematic diagram of the Bag-1 peptide and its deletion mutants. The ubiquitin-like domain is represented in blue and the BAG domain in red. The numbers refer to the amino acid positions of the different domains. The amino acid residues are numbered following the numbering for Bag-1L. B. Prediction of the secondary structure of the Bag-1 peptide, showing an N-terminal β-hairpin from the ubiquitin-like domain (blue) and a C-terminal α-helix from the BAG domain (red). C. Normalized circular dichroism spectra of the Bag-1 peptides. 30 µM of the Bag-1 peptide were measured in 20 mM KHPO_4_ buffer, pH 6.8 (black line). Its α-helical content was estimated to be approximately 25% by deconvolution of the spectra (red line). 12 µM of the N-term peptide (green line) and 11 µM of C-term (blue line) were measured under the same conditions. D. ^1^H^15^N-HSQC NMR spectrum of ^15^N-labeled Bag-1 peptide (202–269) in 20 mm KHPO_4_ buffer, pH 6.8, at 23°C. The narrow spectral dispersion indicates that the peptide does not exhibit a folded globular structure. The H_δ_ and H_ε_ side chain signals of asparagine and glutamine are connected by thin lines. E. The N-terminal region of the Bag-1 peptide is important for GRP78 binding. 400 µg of 22Rv.1 cell lysate were incubated with glutathione-agarose beads carrying 15 µg GST-N-term peptide (lane 2), GST-C-term peptide (lane 3), GST-ΔUbi peptide (lane 4), GST-Bag-1(202–269) peptide (lane 5) and GST (lane 6). The beads were washed and the bound proteins were separated by 10% SDS-PAGE and subjected to Western blotting using antibodies directed against GRP78 or GST. The input lane shows 1/10 aliquot of cell lysate used for the study. F. Clonogenic assay of the Bag-1 peptides expressed in 22Rv.1 cells. Cells transfected with the indicated constructs were selected in medium containing neomycin and the colonies formed were quantified. Shown are the mean value ±SEM of at least three independent experiments using three different plasmid preparations (*p<0.05). G-I. The N-terminal peptide reduces tumor growth *in vivo*. Six-week old athymic nude mice were injected subcutaneously on both flanks with 5×10^6^ cells of each stable clone. Tumor size was measured once per week using a caliper and expressed as tumor volume in mm^3^. Shown are the tumor volumes of clones transfected with the N-terminal peptide (G), the C-terminal peptide (H) and the ΔUbi peptide (I). Each point represents the mean volume and standard deviation of at least 5 to 10 tumors.

**Figure 6:**
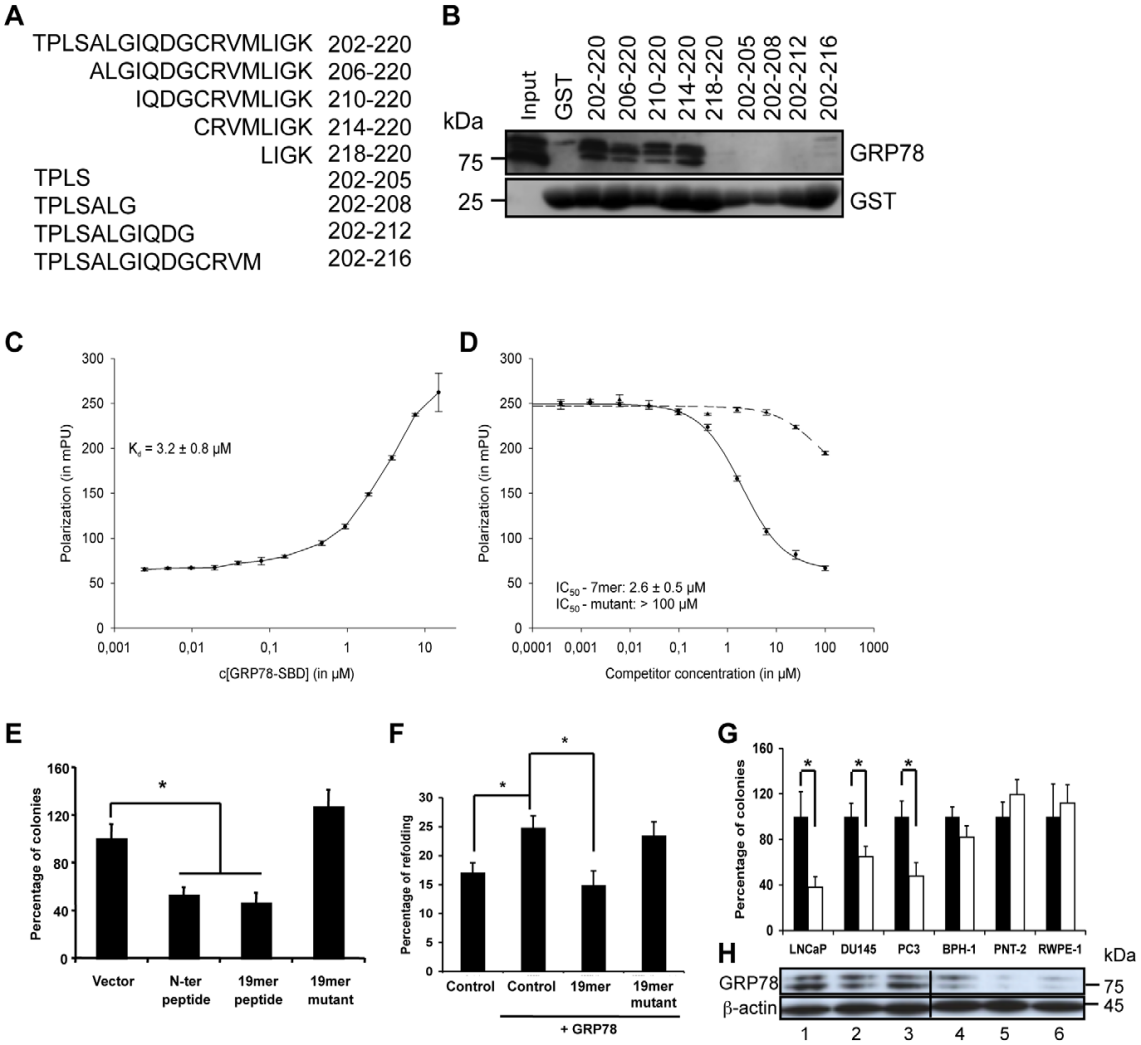
Selectivity of action of the Bag-1 peptide. A Amino acid sequence of the 19mer peptide and its deletion mutants. B GST pull-down assay performed incubating 400 µg of 22Rv.1 cell lysate and 25 µg of GST-fused deletion mutants of the 19mer peptide. After the pull-down experiment, Western blots were carried out with anti-GRP78/BiP and GST antibodies. C. Fluorescence polarization (FP) assays with a 10 nM FITC-labeled 7-mer Bag-1 peptide and dilutions of the GST-fused substrate binding domain of GRP78 in 60 µl of Tris-GSH buffer (50 mM Tris, 10 mM reduced glutathione and 0.1% BSA, pH 8.0). Triplicate samples were measured after 5 h of incubation at room temperature with an Infinite F-200 reader (Tecan) with excitation at 490 nm and emission at 535 nm. Binding constants were determined from three independent experiments using non-linear regression in Sigma Plot 10.0. D. Competition assays with a complex of 10 nM of FITC-labeled Bag-1 7-mer peptide and 0.75 mg/ml GST-fused GRP78 substrate binding domain was measured against a dilution series from 100 µM to 0.1 nM of wild-type Bag-1 7-mer peptide (filled line) or triple mutant peptide (dotted line) in 40 µl of Tris-GSH buffer. FITC-peptide and the FITC-peptide-protein complex were used as controls. Fluorescence polarization was determined as described above and IC_50_ values were determined in Sigma Plot. E. Clonogenic assay in 22Rv.1 cells with pcDNA3 based vectors coding for the N-terminal Bag-1 peptide (positive control), the 19mer and the 19mer mutant fragments as well as the empty expression vector (negative control). Cells were transfected with the indicated constructs and selected in medium containing neomycin. The colonies were stained and quantified. Shown as bar chats are the mean ± SEM of at least three independent experiments with three different plasmid preparations. (*p<0.05). F. *In vivo* luciferase refolding assay was performed in HEK-293 cells transfected at 70% confluency with 2 µg β-actin-fire fly luciferase construct, 3 µg pcDNA3 empty vector as control or pCMV6-GRP78, 6 µg pcDNA3.1-HA-19-mer or 19-mer mutant peptide and 0.5 µg Renilla luciferase construct to determine the transfection efficiency. Shown as bar charts is the mean of at least three different experiments ± SEM (*p<0.05). G. Clonogenic assay with the indicated malignant (LNCaP, DU145, PC3) and benign (BPH1, PNT2, RWPE1) prostate cell lines transfected with the vector control (black bars) and a vector expressing the 19-mer peptide (white bars). Shown are the mean ± SD of at least three different experiments performed with three different plasmid preparations (*p<0.05). H. Western blot analysis of cell lysates derived from the prostate cancer cells used for the colony forming assay with specific antibodies against GRP78 and β-actin.

We stably transfected 22Rv.1 prostate cancer cells with a construct coding for an HA-tagged Bag-1 peptide or an empty vector as control and treated the cells with thapsigargin that induces stress by calcium depletion from the ER [47]. In the 22Rv.1 vector control transfected cells, all three arms of the UPR were activated following treatment with thapsigargin (i.e. increased phosphorylation of PERK, increased eIF2α phosphorylation, enhanced expression of ATF4, increased IRE1α phosphorylation, increased cleavage of ATF6 and increased expression of GRP78/BiP (Figure 3A lanes 1–3). The increase cleavage of ATF6 was not accompanied by a concomitant downregulation of uncleaved ATF6 since thapsigargin enhanced the expression of this gene (Figure S3A). This effect was not only observed in 22Rv.1 cells but also seen in LNCaP prostate cancer cells at the protein level (Figure S3B). The overexpression of the Bag-1 peptide affected all three arms of this pathway. For example, there was reduced ER stress-induced phosphorylation of PERK and IRE1 (Figure 3A lanes 4–6). There was also a significant reduction of cleaved ATF6 (Figure 3A lanes 4–6; Figures S3A and S3B) and an inhibition of the thapsigargin-induced expression of GRP78/BiP (Figure 3A lanes 4–6). Intriguingly an increase in eIF2α phosphorylation was observed leading to activation of the downstream target ATF4 (Figure 3A lanes 4–6). As PERK is downregulated following overexpression of the Bag-1 peptide, it cannot be the kinase responsible for the increased phosphorylation of eIF2α in the peptide expressing cells. It is likely that another kinase is responsible for the increase phosphorylation of eIF2α Indeed we could show that the upstream kinase, general control nonderepressible 2 (GCN2) is upregulated in the peptide expressing cells (Figure S4). The enhanced phosphorylation of eIF2α phosphorylation and activation of ATF4 in the peptide expressing cells are correlated with an increased expression of the pro-apoptotic factor CHOP indicating that the Bag-1 peptide induces apoptosis. Further evidence that the peptide is involved in the induction of apoptosis is shown in experiment in which we treated 22Rv.1 cells with thapsigargin for 24 h and compared the CHOP expression in control cells with cells expressing the Bag-1 peptide. Twenty-four hours treatment resulted in induced CHOP expression accompanied by an increase in PARP cleavage even in the control cells. However the level of CHOP expression and PARP cleavage was further enhanced in the cells expressing the Bag-1 peptide indicating that the peptide sensitizes the cells to the apoptosis inducing effect of thapsigargin. Following siRNA knockdown of CHOP, PARP cleavage was reduced in both cells line indicating that this protein is responsible for the apoptosis inducing effect measured by PARP cleavage (Figure S5).

To determine if stress-induced apoptosis is indeed increased in the presence of the Bag-1 peptide, the 22Rv.1 cells expressing this peptide were treated with thapsigargin or glucose starved to induced ER stress [45] and the expression of apoptotic markers (caspase 4 and PARP) were analyzed. In the absence of the ER stressors, both caspase 4 and PARP cleavage was increased in the 22Rv.1 cells overexpressing the Bag-1 peptide compared to the vector control cell line (Figure 3B compare lane 4 with 1). However in the presence of ER stress, a tremendous increase in caspase 4 expression and PARP cleavage was observed in the Bag-1 peptide expressing cells compared to the control (Figure 3B compare lanes 5–6 with 2–3). These results confirm that the expression of the Bag-1 peptide induces apoptosis as well as sensitizing the prostate tumor cells to stress-induced apoptosis. Further evidence of increased ER apoptosis by the Bag-1 peptide is the enhanced expression of JNK and caspase 7 cleavage in the 22Rv.1 cells expressing the peptide compared to control vector transfected cells in the presence of thapsigargin (Figure S6).

To find out whether the effect of the Bag-1 peptide is correlated with the cellular levels of GRP78/BiP, the level of PARP cleavage was compared in vector and Bag-1 peptide expressing 22Rv.1 cells following the knock-down of GRP78/BiP by siRNA interference technique. Consistent with the finding in Figure 3B, the expression of the Bag-1 peptide led to an increase in the PARP cleavage even in the absence of ER stress (Figure 3C compare lanes 1 and 2). However following the knock-down of GRP78/BiP, PARP cleavage was enhanced in the control 22Rv.1 cells (Figure 3C compare lane 3 with lane 1) but no difference in the cleavage pattern was further observed in the cells expressing the Bag-1 peptide (Figure 3C compare lanes 3 and 4).

### Bag-1 Specific Sequence Inhibits Prostate Tumor Cell Growth

To better analyze the effect of the Bag-1 peptide on prostate cell growth, we determined the proliferation of single 22Rv.1 cell clones expressing either the Bag-1 peptide or an empty expression vector using CellTiter-Blue™ proliferation assay. The peptide clones (P25, P29 and P42), even in the absence of ER stress, showed a significantly reduced proliferation compared to the vector controls (V18, V19 and V33) (Figures 4A and B). When these stably transfected cells were injected into athymic nude mice, cells containing the empty expression vector developed tumors with an average allowable volume of 1.2 cm^3^ in 4–8 weeks (Figure 4C). In contrast, tumors generated by cells expressing the peptide (P42, P25 and P29) only reached 0.4 cm^3^ in 9 weeks (Figure 4D). This difference in tumor volume was also reflected in the weight of the tumors determined at the end of the experiment (Table 1). Note that in some cases (e.g. clone 25), the mice were euthanized earlier than 9 weeks because the tumors were necrotic (Table 1). Similar results on the inhibition of tumor cell growth in athymic mice were obtained when the peptide was stably expressed in the prostate tumor cell line LNCaP (Table 1).

Immunohistological studies were carried out on the tumors generated by the 22Rv.1 cell clones using a TUNEL assay. Consistent with the growth profile, tumors derived from the control clones (V18, V19 and V33) showed very few TUNEL positive cells. In contrast, tumors derived from the peptide-expressing clones (P25, P29 and P42) showed strong signals indicative of massive apoptosis particularly in the cells expressing high levels of the Bag-1 peptide (Figure 4E) confirming a pro-apoptotic action of the Bag-1 peptide even in an *in vivo* situation.

### Structural Studies of the Bag-1 Peptide

The Bag-1 peptide we have shown to inhibit tumor cell growth consists N-terminally of twenty amino acids that form an extended loop and a β-sheet of the ubiquitin-like domain (1WXV.pdb), followed by helix 1 of the BAG domain (1I6Z.pdb) (Figure 5A). We used homology-based structure prediction methods, as detailed in Text S1, to identify key structural features of this sequence. The resulting model, which represents an idealized average structure is shown in Figure 5B.

Structure analysis by circular dichroism (CD) showed that the Bag-1 peptide adopts some residual α-helical structure in about 25% of its sequence (∼17 residues), in agreement with the structure prediction (Figure 5C black line). To identify the degree of structural disorder, i.e. the degree of fluctuation around the average structure, we analyzed ^15^N-HSQC spectra, which showed only little dispersion (Figure 5D). The majority of the amide protons resonated within a narrow range between 7.8 and 8.3 ppm (Figure 5D). No upfield-shifted signals indicative of defined three-dimensional interactions were observed in the 1D spectrum. This suggests that the partial helicity in the CD spectrum does not originate from a well-defined three-dimensional structure, but rather represents a tendency of this segment to fluctuate around a mean helical structure. This interpretation is consistent with the CD estimate of 17 α-helical-residues which is only half of the 33 residues predicted by the model (Figure 5B). We further dissected the peptide into two smaller fragments, a N-terminal 40 residues (N-terminal) and a 28 C-terminal fragment (C-terminal) (Figure 5A). The CD spectra of both fragments fused to an HA-tag for solubility, revealed that in isolation both parts are entirely unstructured as clearly seen from the pronounced minimum at 198 nm and the absence of any positive ellipticity below 200 nm (Figure 5C blue and green lines).

In GST-pull down assay with lysates from the 22Rv.1 cells, we could show that although the peptides are unstructured, the N-terminal but not the C-terminal peptide bound to GRP78/BiP (Figure 5E compare lanes 2 and 5 with lane 3). This interaction is however different from that of the full-length Bag-1 peptide. While Bag-1 or the full length 68 amino acid peptide interacted with both the SBD and ATPase binding domains of GRP78 (Figure 1E and results not shown), the N-terminal peptide interacted preferentially with the SBD (Figure S7). Deletion of the 19 amino acid ubiquitin-like domain portion of the Bag-1 peptide (Figure 5A) abrogated the binding to GRP78 (ΔUbi peptide, Figure 5E lane 4).

The binding activities of the truncated peptides correlated well with their inhibitory action in cell growth assays. Only the full-length Bag-1 peptide and the N-terminal peptide inhibited growth of the 22Rv.1 cells in clonogenic assay while the C-terminal and the ΔUbi peptide were ineffective (Figure 5F). The growth inhibitory action of the peptides was also confirmed in xenograft models using single clones of 22Rv.1 cell stably expressing the N-terminal peptide. This effect was not seen with clones expressing the C-terminal or ΔUbi peptides (Figures 5G-I). These results together show that the sequence that extends into the ubiquitin-like domain of Bag-1 is important for binding to GRP78/BiP and for the inhibition of prostate tumor cell growth.

### Further Truncation of the Bag-1 Peptide

Further N- and C-terminal truncations of the 19 amino acid peptide (19mer) (Figure 6A) led to the identification of a seven amino acid core peptide ^214^RVMLIGK^220^ as important for the binding to GRP78/BiP (Figure 6B). This 7 amino acid core sequence labeled with FITC bound the SBD of GRP78/BiP with a K_D_ of 5.7±0.8 µM as determined in a fluorescence polarization experiment (Figure 6C). Competition experiments with an unlabeled core sequence produced an IC_50_ of 2.6±0.5 µM while a sequence with an exchange of the “VML” in the 7 amino acid sequence with alanine residues (^214^RAAAIGK^220^) was unable to compete for binding (IC_50_ of >100 µM) (Figure 6D). This mutated core when introduced into the 202–220 amino acid peptide (19-mer mutant) significantly reduced the ability of the 19 amino acid sequence to inhibit 22Rv.1 cell growth in the clonogenic assay compared to the unmutated 19 mer (202–220) or the N-terminal peptide (202–241) (Figure 6E). The 19-mer peptide but not the 19-mer mutant peptide also inhibited the ability of GRP78/BiP to refold denatured protein in the *in vivo* luciferase refolding assay (Figure 6F).

To determine the selectivity of the Bag-1 peptide toward inhibition of prostate cancer cell growth, we overexpressed the 19-mer in a series of benign prostate cells and prostate cancer cell lines and performed clonogenic assays with these cells. We could show that the peptide inhibited growth of the prostate tumor cell lines (LNCaP, DU145 and PC3) that express substantial levels of GRP78 (Figures 6G and 6H). In contrast, the benign prostate cell lines BPH-1, PNT-2 and RWPE-1 that marginally express this molecular chaperone were not growth inhibited by the peptide (Figures 6G and 6H).

These results together with our previous results in Figure 3C show that the cell growth inhibitory action of the Bag-1 peptide is correlated with the cellular levels of GRP78/BiP.

## Discussion

GRP78/BiP is expressed in many human cancers where it mediates tumor growth by enhancing proliferation, protecting against apoptosis and promoting tumor angiogenesis. [48]. GRP78/BiP also favors cell survival and contributes to tumor progression and drug resistance during ER stress that arises in the tumor microenvironment as a result of hypoxia and nutrient deprivation [15], [49]. The maintenance of cellular homeostasis by GRP78/BiP occurs in different tumors including prostate cancer [50], [51], [52] and an increased expression of GRP78 has been associated with castration resistance and androgen deprivation in prostate cancer [50]. As a result, several attempts have been made to target GRP78/BiP to trigger apoptosis in prostate cancers and other forms of cancers. For example, a peptidic ligand of GRP78/BiP fused to a programmed cell death-inducing sequence was shown to suppress tumor growth in xenograft and isogenic models of prostate and breast cancer [53]. Furthermore a peptidic ligand of GRP78/BiP conjugated to taxol has been shown to exhibit selective cytotoxicity against highly metastatic melanoma cells [54]. In addition to peptide-drug conjugates, a number of peptides and antibodies binding to the ATPase and substrate binding domains of GRP78/BiP have been reported [55], [56], [57]. Some of these affect the growth promoting and angiogenic action of GRP78/BiP positively or negatively but their modes of action have not been extensively investigated.

In the present study we made use of the ability of the co-chaperone Bag-1 to bind GRP78/BiP to inhibit its refolding activity to derive a Bag-1-based peptide for suppressing the growth promoting action of GRP78/BiP. Our peptide interacts with the C-terminal substrate binding domain of GRP78/BiP, a region bound by an antibody that exerts pro-apoptotic function [58] identifying the C-terminal region as a target for the elicitation of apoptosis by GRP78/BiP.

The Bag-1 peptide we identified inhibited the refolding action of GRP78/BiP and all three arms of the UPR since IRE1α and PERK phosphorylation were inhibited as well as ATF6 cleavage. However it activated the phosphorylation eIF2α, a downstream effector of PERK indicating that this peptide also affects the action of other regulators of eIF2α phosphorylation such as GCN2 [45], [59]. Indeed we could show enhanced phosphorylation of GCN2 in cells expressing the Bag-1 peptide (Figure S4). Alternatively the Bag-1 peptide may increased phosphorylation of eIF2α through inhibition of the action of GADD34, a cellular stress response protein that binds protein phosphatase 1 (PP1) that dephosphorylates eIF2α. Indeed we have observed that the Bag-1 peptide interact with GADD45 (results not shown) in agreement with the reports that Bag-1 binds GADD34 and inhibits the activity of PP1 [32]. Thus the Bag-1 peptide we have identified targets other proteins in addition to GRP78/BiP to induce apoptosis. The enhanced phosphorylation of eIF2α we see in the presence of the Bag-1 peptide is correlated with enhanced expression of the downstream targets ATF4 and CHOP [60], [61]. As a consequence, the cells are more sensitive to stressors such as thapsigargin or glucose starvation, as shown by the increase in casepase 4 activity and PARP cleavage. We could also show that the Bag-1 peptide decreases tumor cell growth *in vivo* in two models of tumor xenografts (22Rv.1 and LNCaP).

The Bag-1 peptide that we found to inhibit prostate tumor cell growth has a length of 68 amino acids, comprising helix 1 of the BAG domain at its C-terminus and another twenty amino acids at its N-terminus from the ubiquitin-like domain. We could demonstrate that an N-terminal 40 amino acid fragment of this peptide lacking the C-terminal BAG domain is still able to inhibit growth. We could further narrow this peptide to 19 amino acids and we identified the sequence RVMLIGK as the core for binding to GRP78/BiP and for the inhibition of prostate tumor growth. This sequence is very hydrophobic and agrees with earlier studies that reported that peptides that bound the Hsp70 chaperones including GRP78/BiP are short, at least 7 amino acids long, and are enriched in large hydrophobic and aromatic residues [62]. The fact that GRP78/BiP plays important roles in maintaining cellular homeostasis and is overexpressed in different tumors [50], [51], [52] makes it very likely that the peptide we have identified may not only be important for the inhibition of prostate tumor growth but could also be used for growth inhibition of a broad range of other tumors. In addition, based on the evidence that cancer cells develop drug resistance by inducing the UPR, the Bag-1 peptide could be used to overcome this problem and to potentiate the anti-tumorigenic effect of already existing drugs [63], [64].

In addition to being an ER resident protein, recent studies have shown that ER stress actively promotes relocalization of GRP78/BiP on cell surface and ectopic expression also causes cell surface relocalization in the absence of ER stress [65]. Multiple domains of GRP78/BiP including the C-terminal substrate binding domain shown in this work to bind the Bag-1 peptide are extracellularly exposed [65]. Our finding that the Bag-1 peptide interacts with the substrate-binding domain of GRP78/BiP further opens the possibility for external application of this peptide for tumor therapy.

## Supporting Information

Figure S1 **Colocalization of Bag-1 with the endoplasmic reticulum.** Immunofluorescence experiment was performed with 22Rv.1 cells fixed with 4% paraformaldehyde. After fixation, cells were permeabilized with a solution of PBS (phosphate buffer saline) containing 0.1% triton-X-100 and blocked with 4% goat serum in PBS. Endogenous Bag-1 (green channel) and the endoplasmic reticulum (red channel) were stained respectively with a Bag-1 antibody (F-7, Santa Cruz, Heidelberg, Germany) and the ER-tracker (Invitrogen, Karlsruhe, Germany). The orange/yellow color indicates co-localization. Images were aquired with a Leica TCS SPE confocal microscope (Software: Leica Application Suite Advance Fluorescence –2.0.1 build 2043– Leica Microsystems, Wetzlar, Germany). The bar represents 25 µm.
(TIF)

Figure S2 **The Bag-1 peptide does not impair ATPase activity of GRP78.** *In vitro* ATPase activity assay of GRP78. A. ATPase activity was measured with the ATPase assay kit from Innova Bioscience (Cambridge, UK). The reaction mixture contained GRP78 (0.5 µg) increasing amounts of GST purified Bag-1 (continuous line) or Bag-1 peptide (dashed line) up to 1.5 µg. The reaction was carried out for 1 h at 37°C with purified ATP according to the manufacturer’s instructions. Each point represents the mean value of three independent experiments ± SEM. B. The purity of protein preparation used for the assay. Shown are 5 µg of purified GRP78 (StressMarq Biosciences, Victoria, Canada), GST Bag-1 peptide, and GST-Bag-1 used in the assay following SDS PAGE and Coomassie blue staining.
(TIF)

Figure S3 **ATF6 expression is upregulated by thapsigargin treatment.** A. Real time PCR analysis of ATF6 gene expression following thapsigargin (300 nM) treatment for the indicated time points in 22Rv.1 cell clones with empty vector control (open bars) and peptide-expressing clones (filled bars). The RNA was extracted using PeqGold RNA pure (PeqLab, Germany) kit according to manufacturer’s instructions. For gene expression analysis, the following primers were used: Rib36 forward 5′-GAAGGCTGTGGTGCTGATGG-3′; reverse 5′-CCGGATATGAGGCAGCAG-3′; ATF6 forward 5′-TTCTTTGGCTCCCCTCCCGCA-3′; reverse 5′-AGTCTGGCAGGGTCCCACGC-3′. Each bar represents the mean of three independent experiments ± SEM. *p<0.05. B. Western blot analysis of ATF6 and its cleaved product (cATF6) in LNCaP cells stably expressing the empty vector control or the Bag-1 peptide. Anti-ATF6 specific antibody and an anti-HA specific antibody were used for the Western blot. β-actin antibody was used to determine equal loading control. Mouse monoclonal anti-ATF6 antibody was purchased from Imgenex (Hamburg, Germany), mouse monoclonal anti-HA antibody (HA.11 clone 16B12) was purchased from Covance (Munich, Germany) and anti-β-actin antibody was purchased from Abcam (Cambridge, UK).
(TIF)

Figure S4 **The Bag-1 peptide induces GCN2 phosphorylation.** 22Rv.1 cells stably expressing the empty vector control or the Bag-1 peptide were treated with thapsigargin (300 nM) for 16 h and subjected to Western blot analysis using anti-phospho-GCN2, GCN2 and HA-specific antibodies. The filters used in this experiment for the GCN2 signals are the same filters used in Figure 3A. The HA-Bag-1 peptide and the β-actin signals are therefore identical to that of Figure 3A.
(TIF)

Figure S5 **Knock-down of CHOP impairs the Bag-1 peptide-mediated increase in apoptosis.** The action of the Bag-1 peptide is dependent on CHOP. Pooled clones of 22Rv.1 expressing an empty expression vector (lane 1 to 4) or an HA-tagged Bag-1 peptide (lane 5 to 8) were transfected with control GFP siRNA (lane 1–2 and 5–6) or CHOP siRNA (lane 3–4 and 7–8). After treatment with 300 nM thapsigargin (TG) for 24 h, cells were lysed and Western blot was carried out with anti-PARP, anti-CHOP and anti-HA antibodies. β-actin antibody was used to determine the level of protein loaded on the gel.
(TIF)

Figure S6 **Overexpression of the Bag-1 peptide increases Capase-7 cleavage and JNK expression.** 22Rv.1 cells stably transfected with an empty vector control (lane 1–2) or a plasmid encoding an HA-tagged Bag-1 peptide (lane 3–4) were treated with 300 nM thapsigargin (TG) for 24 h and harvested for Western blot anaysis. Specific antibodies against JNK (Cell Signaling, Frankfurt am Main, Germany), cleaved Caspase-7 (Cell Signaling, Frankfurt am Main, Germany), HA (HAA.11 clone 16B12, Covance, Munich, Germany) or β-actin (Sigma, Steinheim, Germany) were used in the experiment.
(TIF)

Figure S7 **The N-terminal Bag-1 peptide interacts with GRP78(SBD).** The N-terminal peptide binds to the SBD of GRP78. GST-pull down assay was performed using 100 µg of cell lysate from HEK 293 cells transfected with a plasmid expressing an HA-tagged N-ter-Bag-1 peptide together with 10 µg of the indicated GST-fused protein. After the pull-down experiment, Western blotting was performed with an anti-HA antibody to detect the peptide. Shown is a Commassie blue staining of the bacterially purified GST proteins to demonstrate equal loading of the gel.
(TIFF)

Text S1 **Homology-basded structure prediction of the Bag-1 peptide.**
(DOCX)
