# Supplementary figures and images for: A Peptidic Unconjugated GRP78/BiP Ligand Modulates the Unfolded Protein Response and Induces Prostate Cancer Cell Death
Source: PLoS One. 2012 Oct 1;7(10):e45690. doi: 10.1371/journal.pone.0045690 (PMC3462190; doi:10.1371/journal.pone.0045690)

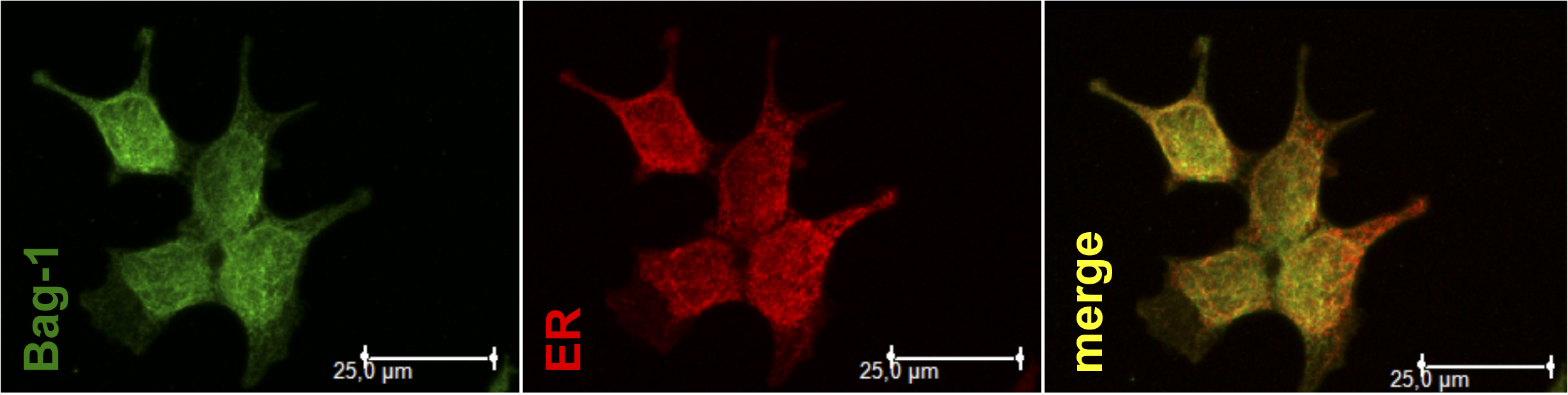

Supplement: Figure S1 — Colocalization of Bag-1 with the endoplasmic reticulum. Immunofluorescence experiment was performed with 22Rv.1 cells fixed with 4% paraformaldehyde. After fixation, cells were permeabilized with a solution of PBS (phosphate buffer saline) containing 0.1% triton-X-100 and blocked with 4% goat serum in PBS. Endogenous Bag-1 (green channel) and the endoplasmic reticulum (red channel) were stained respectively with a Bag-1 antibody (F-7, Santa Cruz, Heidelberg, Germany) and the ER-tracker (Invitrogen, Karlsruhe, Germany). The orange/yellow color indicates co-localization. Images were aquired with a Leica TCS SPE confocal microscope (Software: Leica Application Suite Advance Fluorescence –2.0.1 build 2043– Leica Microsystems, Wetzlar, Germany). The bar represents 25 µm. (TIF) [file pone.0045690.s001.tif]

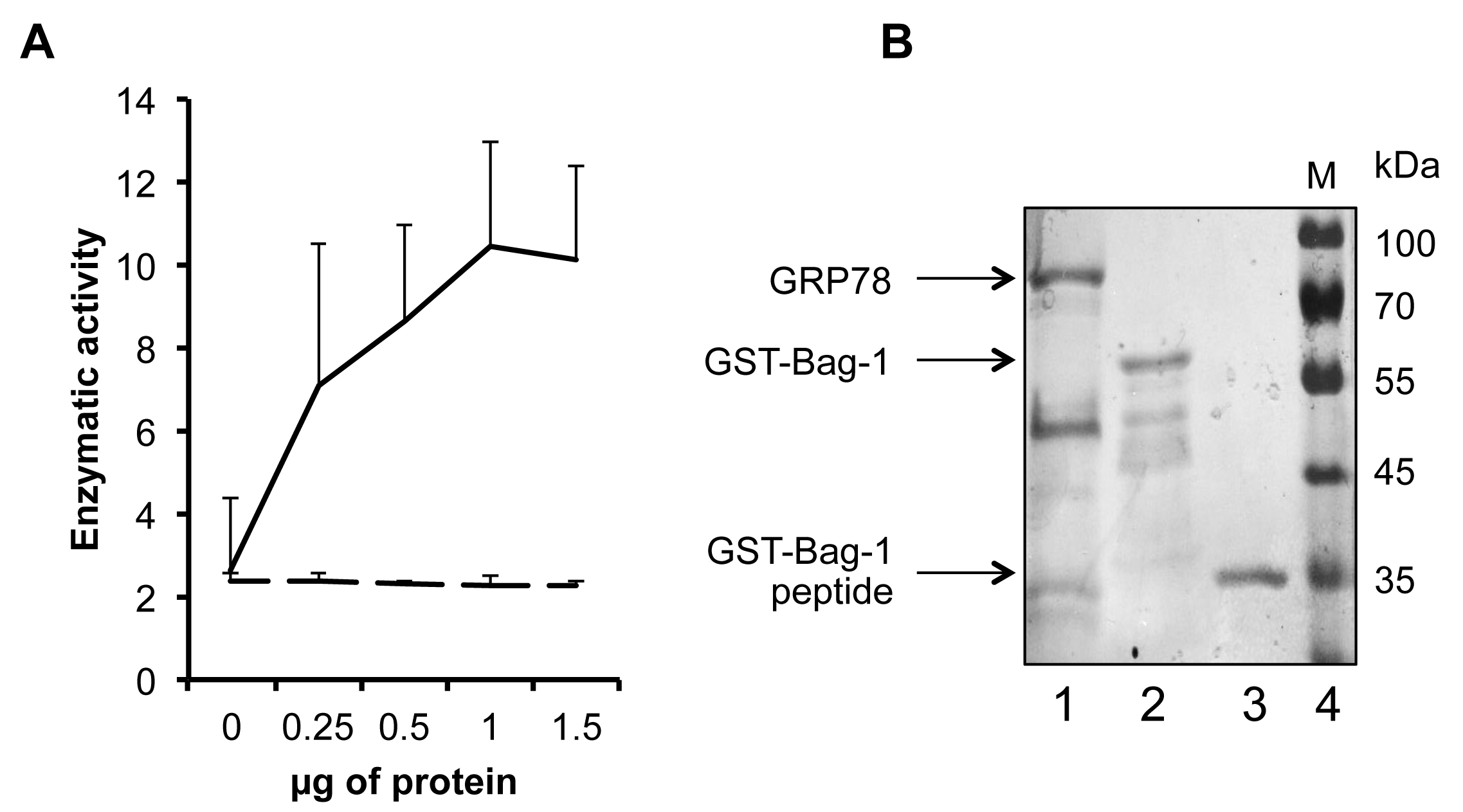

Supplement: Figure S2 — The Bag-1 peptide does not impair ATPase activity of GRP78. In vitro ATPase activity assay of GRP78. A. ATPase activity was measured with the ATPase assay kit from Innova Bioscience (Cambridge, UK). The reaction mixture contained GRP78 (0.5 µg) increasing amounts of GST purified Bag-1 (continuous line) or Bag-1 peptide (dashed line) up to 1.5 µg. The reaction was carried out for 1 h at 37°C with purified ATP according to the manufacturer’s instructions. Each point represents the mean value of three independent experiments ± SEM. B. The purity of protein preparation used for the assay. Shown are 5 µg of purified GRP78 (StressMarq Biosciences, Victoria, Canada), GST Bag-1 peptide, and GST-Bag-1 used in the assay following SDS PAGE and Coomassie blue staining. (TIF) [file pone.0045690.s002.tif]

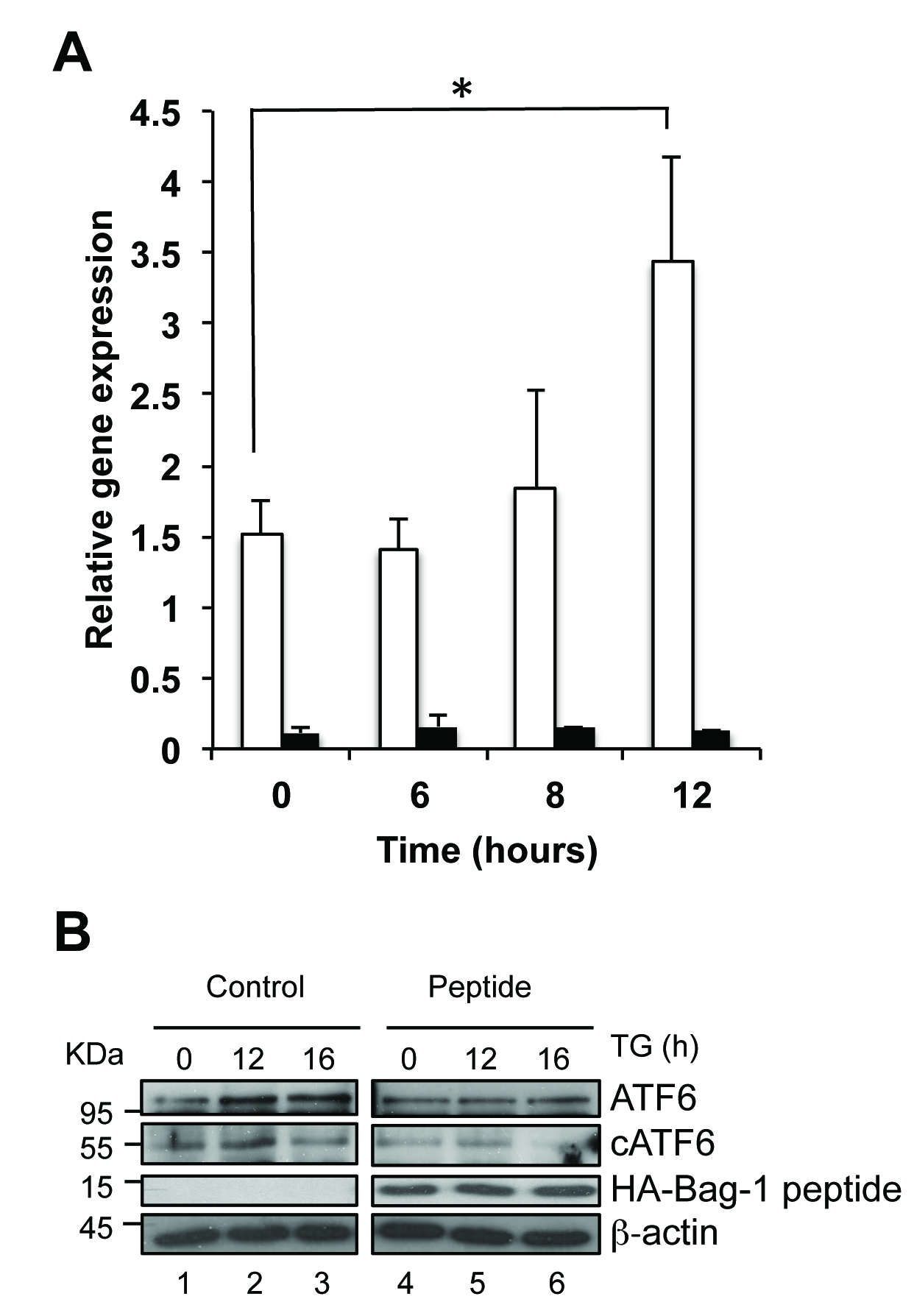

Supplement: Figure S3 — ATF6 expression is upregulated by thapsigargin treatment. A. Real time PCR analysis of ATF6 gene expression following thapsigargin (300 nM) treatment for the indicated time points in 22Rv.1 cell clones with empty vector control (open bars) and peptide-expressing clones (filled bars). The RNA was extracted using PeqGold RNA pure (PeqLab, Germany) kit according to manufacturer’s instructions. For gene expression analysis, the following primers were used: Rib36 forward 5′-GAAGGCTGTGGTGCTGATGG-3′; reverse 5′-CCGGATATGAGGCAGCAG-3′; ATF6 forward 5′-TTCTTTGGCTCCCCTCCCGCA-3′; reverse 5′-AGTCTGGCAGGGTCCCACGC-3′. Each bar represents the mean of three independent experiments ± SEM. *p<0.05. B. Western blot analysis of ATF6 and its cleaved product (cATF6) in LNCaP cells stably expressing the empty vector control or the Bag-1 peptide. Anti-ATF6 specific antibody and an anti-HA specific antibody were used for the Western blot. β-actin antibody was used to determine equal loading control. Mouse monoclonal anti-ATF6 antibody was purchased from Imgenex (Hamburg, Germany), mouse monoclonal anti-HA antibody (HA.11 clone 16B12) was purchased from Covance (Munich, Germany) and anti-β-actin antibody was purchased from Abcam (Cambridge, UK). (TIF) [file pone.0045690.s003.tif]

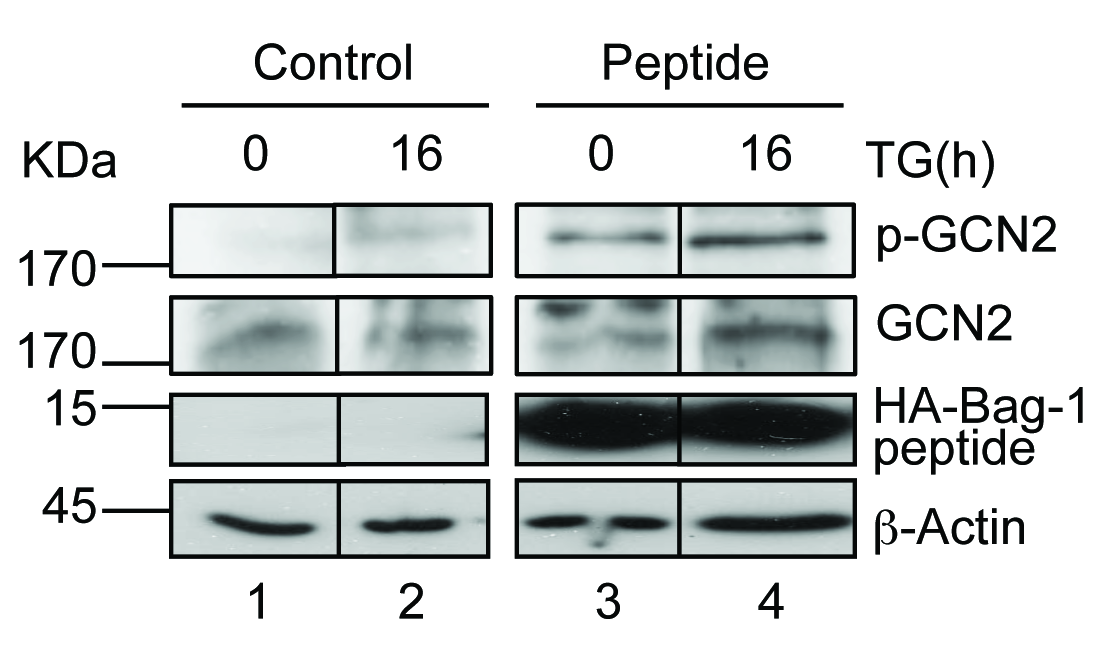

Supplement: Figure S4 — The Bag-1 peptide induces GCN2 phosphorylation. 22Rv.1 cells stably expressing the empty vector control or the Bag-1 peptide were treated with thapsigargin (300 nM) for 16 h and subjected to Western blot analysis using anti-phospho-GCN2, GCN2 and HA-specific antibodies. The filters used in this experiment for the GCN2 signals are the same filters used in Figure 3A. The HA-Bag-1 peptide and the β-actin signals are therefore identical to that of Figure 3A. (TIF) [file pone.0045690.s004.tif]

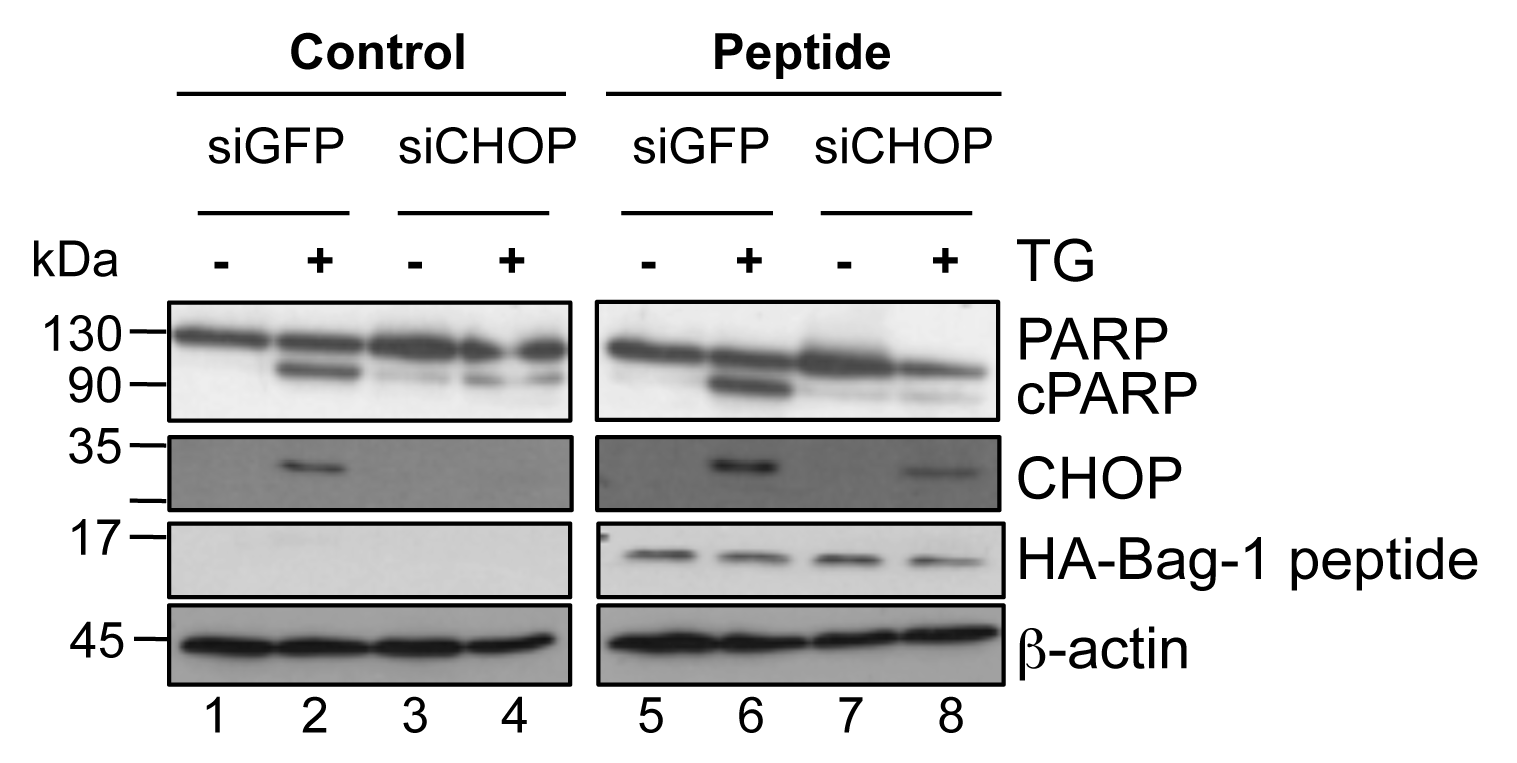

Supplement: Figure S5 — Knock-down of CHOP impairs the Bag-1 peptide-mediated increase in apoptosis. The action of the Bag-1 peptide is dependent on CHOP. Pooled clones of 22Rv.1 expressing an empty expression vector (lane 1 to 4) or an HA-tagged Bag-1 peptide (lane 5 to 8) were transfected with control GFP siRNA (lane 1–2 and 5–6) or CHOP siRNA (lane 3–4 and 7–8). After treatment with 300 nM thapsigargin (TG) for 24 h, cells were lysed and Western blot was carried out with anti-PARP, anti-CHOP and anti-HA antibodies. β-actin antibody was used to determine the level of protein loaded on the gel. (TIF) [file pone.0045690.s005.tif]

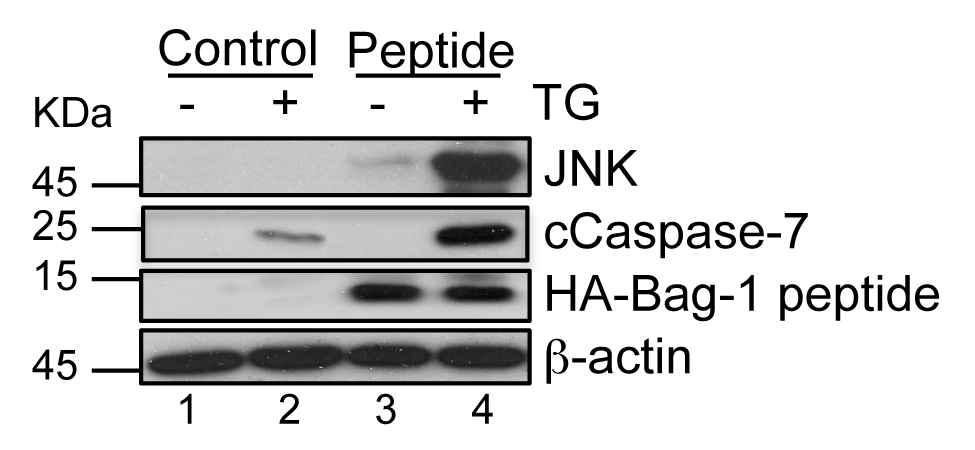

Supplement: Figure S6 — Overexpression of the Bag-1 peptide increases Capase-7 cleavage and JNK expression. 22Rv.1 cells stably transfected with an empty vector control (lane 1–2) or a plasmid encoding an HA-tagged Bag-1 peptide (lane 3–4) were treated with 300 nM thapsigargin (TG) for 24 h and harvested for Western blot anaysis. Specific antibodies against JNK (Cell Signaling, Frankfurt am Main, Germany), cleaved Caspase-7 (Cell Signaling, Frankfurt am Main, Germany), HA (HAA.11 clone 16B12, Covance, Munich, Germany) or β-actin (Sigma, Steinheim, Germany) were used in the experiment. (TIF) [file pone.0045690.s006.tif]

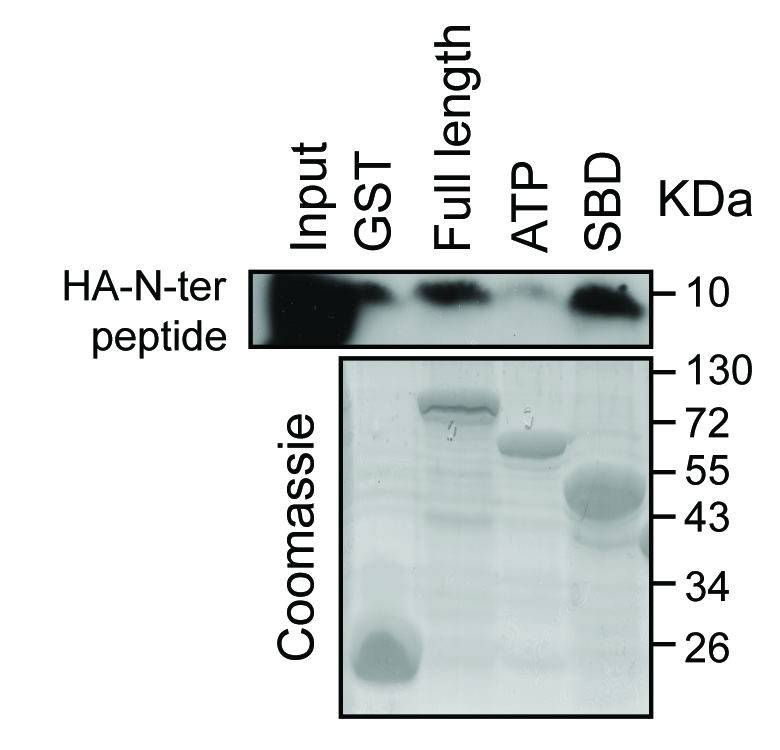

Supplement: Figure S7 — The N-terminal Bag-1 peptide interacts with GRP78(SBD). The N-terminal peptide binds to the SBD of GRP78. GST-pull down assay was performed using 100 µg of cell lysate from HEK 293 cells transfected with a plasmid expressing an HA-tagged N-ter-Bag-1 peptide together with 10 µg of the indicated GST-fused protein. After the pull-down experiment, Western blotting was performed with an anti-HA antibody to detect the peptide. Shown is a Commassie blue staining of the bacterially purified GST proteins to demonstrate equal loading of the gel. (TIFF) [file pone.0045690.s007.tif]
