# Supplementary material for: A Peptidic Unconjugated GRP78/BiP Ligand Modulates the Unfolded Protein Response and Induces Prostate Cancer Cell Death
Source: PLoS One. 2012 Oct 1;7(10):e45690. doi: 10.1371/journal.pone.0045690 (PMC3462190; doi:10.1371/journal.pone.0045690)
Supplement: Text S1 — Homology-basded structure prediction of the Bag-1 peptide. (DOCX) [file pone.0045690.s008.docx]

**Supplementary text (Text S1)**

**Structural studies of the Bag-1 peptide**

We used 3D jury [[1](#_ENREF_1)] to find low-homology consensus alignments to the Bag-l sequence. We found a significant overlap with regions of five different proteins, three of which are chaperones or chaperone inhibitors of the BAG domain.


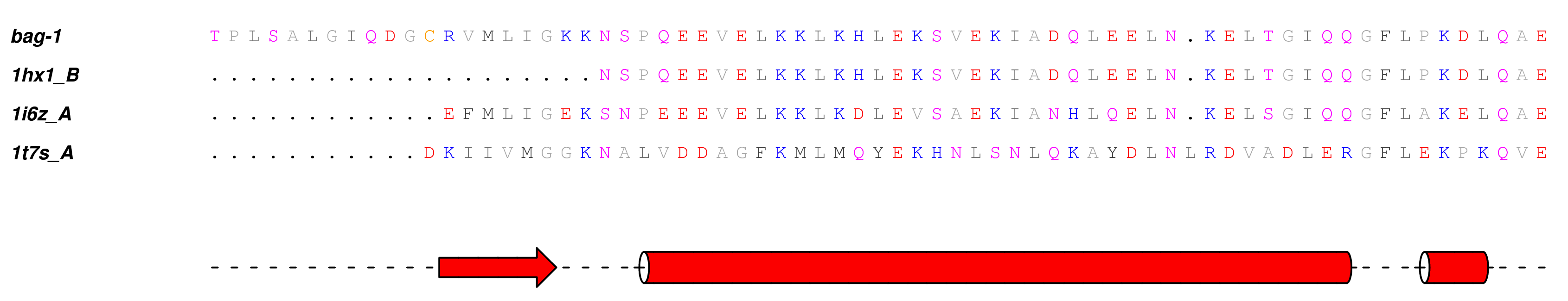


**Alignment of the Bag-1 sequence with the three homologous proteins identified by 3D jury.** This figure was prepared using ALINE [[2](#_ENREF_2)]. Out of the three we chose 1t7s_A as the template, since its alignment extends furthest into the N-terminal region. All three sequences adopt a helix-turn-helix motif in the region 23-65. In addition, the secondary structure prediction of the Bag-l sequence by PSIPRED and PROFSEC [[3](#_ENREF_3)] indicates an extended (β-sheet) region between residues R13 and G18, where template 1t7s_A has a β-sheet motif in the region with Bag-l. Further the FFAS03 [[4](#_ENREF_4)] server ranks template 1t7s_A with greater confidence than the other two templates. We therefore used the FFAS03 alignment obtained via the 3D jury interface for further modeling. We modeled the structure with FFAS03 alignment with 1t7s_A as template using Swiss-Pdbviewer which uses SWISS-MODEL [[5](#_ENREF_5)] for comparative modeling. The resulting model was subjected to energy minimization using a simulated annealing (SA) protocol with POEM using the PFF02 [[6](#_ENREF_6)] free energy force field. The stability of the structure was tested in 20 basin-hopping simulations (each consisting of 100 SA cycles) on the minimized structure. 7 out 10 lowest energy structures of basin-hopping simulations preserve the secondary structure motif of the initial model, though the tertiary structure is changed in the N-terminal region where the β-hairpin motif shifts to the opposite side of the C-terminal helix.

**References**

1. Ginalski K, Elofsson A, Fischer D, Rychlewski L (2003) 3D-Jury: a simple approach to improve protein structure predictions. Bioinformatics 19: 1015-1018.

2. Bond CS, Schuttelkopf AW (2009) ALINE: a WYSIWYG protein-sequence alignment editor for publication-quality alignments. Acta Crystallogr D Biol Crystallogr 65: 510-512.

3. Jones DT (1999) Protein secondary structure prediction based on position-specific scoring matrices. J Mol Biol 292: 195-202.

4. Jaroszewski L, Rychlewski L, Li Z, Li W, Godzik A (2005) FFAS03: a server for profile--profile sequence alignments. Nucleic Acids Res 33: W284-288.

5. Guex N, Peitsch MC (1997) SWISS-MODEL and the Swiss-PdbViewer: an environment for comparative protein modeling. Electrophoresis 18: 2714-2723.

6. Verma A, Wenzel W (2009) A free-energy approach for all-atom protein simulation. Biophys J 96: 3483-3494.
